# Assessment of the Dietary Intake of High-Rank Professional Male Football Players during a Preseason Training Week

**DOI:** 10.3390/ijerph17228567

**Published:** 2020-11-18

**Authors:** Anna Książek, Aleksandra Zagrodna, Małgorzata Słowińska-Lisowska

**Affiliations:** Department of the Biological and Medical Basis of Sport, University School of Physical Education, 51612 Wrocław, Poland; aleksandra.zagrodna@awf.wroc.pl (A.Z.); malgorzata.slowinska-lisowska@awf.wroc.pl (M.S.-L.)

**Keywords:** diet, sports nutrition, team-based sport, athletes, periodized nutrition, soccer

## Abstract

A well-balanced diet is one of the main factors that may play a supportive role in enhancing acute training stimuli in optimal training adaptation. The aim of the present study was to examine the energy and macro- and micronutrient intake including and excluding supplements among top-level Polish football players during one week of the general preparatory period. In addition, the study looked at whether athletes consume carbohydrates in recommended amounts, depending on the completed training sessions. A total of 26 professional football players were included in the study. The preseason dietary intake was assessed using a 7-day estimated food record. The energy value of the diet and the amounts of the dietary ingredients were assessed using the software Dieta 6.0. The average consumption of energy, vitamin B_2_, vitamin C, vitamin E, folate, and calcium was lower than recommendations, and average intake of sodium and potassium was higher than the norm in the diets of the athletes. The results of this study do not confirm the justification for adding protein preparations to diets of the studied players. Furthermore, football players dietary carbohydrate intake was relatively low in comparison to requirements based on training loads. Based on our results we conclude that further work is necessary to reinforce education about nutritional habits and adjust nutritional strategies to individual needs to enhance athletic performance.

## 1. Introduction

Football is currently one of the most popular sports disciplines worldwide. Whatever the origin and geographic region, professional football players are required to possess a wide range of skills, such as speed, stamina, flexibility, dribbling skill, and the ability to make quick decisions during a match. Furthermore, the physiological demands of this sport are based on running moderate to long distances and engaging in high intensity bouts of movement with variable activity patterns and short bouts of rest. Football players use both anaerobic and aerobic systems to fuel performance. Therefore, players depending on the nature of training, match-playing, and periods of training, will have unique energy demands and nutrient requirements [1,2,3].

It should be noted that adaptations, initiated by exercise, can be amplified or reduced by nutrition. A well-balanced diet plays a key role in proper recovery and enhancing performance, but so too do changes in nutritional intake in response to certain periods of training, so-called nutrition periodization [4]. Periodized nutrition supports training levels and requirements for energy and macronutrients demands throughout a season and/or training period [5].

The emphasis on physical preparation and the increasing competition in football significantly increases the demand for macro- and micronutrients. This requirement can generally be met by a well-balanced diet without the need for dietary supplements. However, the increase in popularity of supplements results in athletes more commonly using such products [6,7]. In consideration of this, the nutritional balance should also take into account the food supplements and foodstuffs intended for particular nutritional uses by football players. Despite the increased interest in nutrition and use of dietary supplements to enhance performance, some athletes might be consuming diets that are less than optimal.

A meta-analysis of the literature from 2000 to 2019 evaluated publications reporting on a total of 277 players playing at elite or sub-elite levels from 12 countries around the world. It showed irregularities in the supply of macronutrients: proteins and carbohydrates. The authors of the research suggest that an inadequate diet may have a significant impact on the health and exercise capacity of players [3]. The literature contains little data on the evaluation of the dietary intake of top-level Polish football players. Therefore, the aim of the present study was to examine the energy and macro- and micronutrient intake including and excluding supplements among high-rank professional Polish football players during one week of the general preparation period. In addition, we verified whether athletes consume carbohydrates in recommended amounts, depending on the completed training sessions. Furthermore, we checked whether the players met the demand for macronutrients, vitamins, and minerals during the training period, which is characterized by long/high-intensity workouts that increase the need for nutrients. The proper supply of nutrients is extremely important in supporting the training process, adaptions to exercises, and optimal preparation of players for the competition period.

## 2. Materials and Methods

### 2.1. Participants

Twenty-six football players (land fielders: 8 defenders, 14 midfielders, and 4 strikers; goalkeepers were excluded because of a different training schedule) were recruited from one club competing in the highest male football Polish league, the “Ekstraleague”. Athletes who had submitted incomplete dietary records and those with injuries/long-term injuries were excluded from the study. The participants’ characteristics are shown in Table 1. All the players were active participants in the summer general preparation period and had similar training loads (Table 2). The competitors who participated in the study were a very homogeneous group, characterized by a similar level of athletic performance, career duration, and applied training loads. It should also be emphasized that the consumption of products and dietary supplements was similar.

All subjects gave their informed consent for inclusion before they participated in the study. The study was conducted in accordance with the Declaration of Helsinki, and the protocol was approved by the Bioethics Committee of the University School of Physical Education, Wrocław, Poland, resolution number 18/2013.

### 2.2. Measurements

Measurements were taken in a fasting state, and the competitors were measured in their underwear, without footwear and socks. Athletes’ height and body mass were measured twice (Day 1 and Day 6) by means accurate to within 0.1 cm and 0.1 kg, respectively. The final results were computed on the basis of the arithmetic average of the measurements. Height was measured with an anthropometer and body mass was measured with an electronic scale.

Body composition (body fat, fat free body mass (FFM), total body water (TBW), muscle mass) was determined using the single frequency bioelectric impedance analyzer (BIA, 50-kHz) manufactured by Akern Bioresearch (Italy).

Aerobic performance (VO_2max_/VO_2peak_) were assessed using a 20 m multistage shuttle running field-test [8]. This method most closely reflects the football effort due to the environment in which physical effort is implemented [9,10,11].

### 2.3. Dietary Intake

Dietary intake was evaluated using a food record method (foods and beverages consumed over 24 h for 7 consecutive days, including one day with no training). The food diary was kept during one week of the preparation period. The athletes were instructed on how to fill in the food record. The authors of the study tried to minimize the risk of improperly completed nutrition logs by instructing the athletes on how to fill them out correctly. In addition, the authors of the study were in constant contact with players. The participants self-reported all the consumed foods and fluids and provided information about the times, types, and portion size of the meals and beverages they consumed. The quantities of all the food/beverage items were reported in household measures or, if available, according to packaging details. If the answers provided in the assigned charts of the 7-day dietary food records were unclear or illegible, the athletes were asked further questions on the spot so as to ensure the maximum accuracy of responses relating to product consumption. The energetic value of food intake, as well as the ingredients of products and meals, were estimated by means of the Dieta 6.0 software (IŻŻ, Warsaw, Poland). Dieta 6.0 is a valuable application that calculates the intake of different nutrients, with bioavailability taken into account. Additionally, athletes were asked to report whether they used any supplements. Moreover, studied players reported the exact amount of dietary supplements consumed within the one week of the preparatory period. Therefore, each diet was calculated in two variants: excluding the supplementation or including the supplementation. Players used the following food supplements: carbohydrate, creatine, energy gels, isotonic drinks, multivitamins, sports bars, and whey protein isolates. A summary of the supplements used is given in Table 3.

The consumption of energy minerals and vitamins was compared against the recommended norms for athletes according to Benardot [12]. The consumption of macronutrients was compared against the recommended norms of the Academy of Nutrition and Dietetics (AND), the Dietitians of Canada (DC), and the American College of Sports Medicine (ACSM) [13].

### 2.4. Statistical Analysis

The statistical analysis was performed using STATISTICA version 10 (2011) from StatSoft, Inc. Parametric tests (the *t*–Student test) were used for normally distributed variables and non–parametric tests (the Wilcoxon signed-rank test) for variables that did not meet the criterion for normal distribution. The level of statistical significance was set at *p* ≤ 0.05.

## 3. Results

Table 2 presents the comparison of average carbohydrate intake of the studied athletes and recommended carbohydrate intake. The results showed that the average daily carbohydrate consumption was 4.7 ± 1.4 and 5.4 ± 1.5 g/kg body mass/day on Days 1 and 4, respectively, i.e., the days when the athletes performed two training units. Almost 77% and 53% of the athletes did not meet their carbohydrate needs on the days with two training units (Day 1 and Day 4). The average carbohydrate consumption on the days when athletes were performing a single training unit, Days 2, 3, and 5, was 5.3 ± 13, 4.8 ± 1.4, and 5.2 ± 1.5 g/kg body mass/day, respectively. The results showed that only 42.3%, 53.8%, and 53.8%, respectively, covered the demand for this component. On the highest-intensity training day (Day 6), the average carbohydrate consumption was 6.1 ± 1.8 g/kg body mass/day. It was found that 42.3% of the athletes did not meet the consumption norm for this ingredient. On a training-free day, the average carbohydrate consumption was 5.3 ± 1.5 g/kg body mass/day. Only 26.9% failed to meet the demand for carbohydrates, and 50% of them exceeded the recommended dose.

The average of energy and macronutrient intake including and excluding supplements is shown in Table 4. The mean consumption of all the dietary components was significantly higher after taking into account all the food supplements and foodstuffs intended for particular nutritional uses.

The mean energy content in the diet of those football players who were not taking food supplements and those who were taking food supplements and foodstuffs intended for particular nutritional uses was 2480.3 ± 388.6 kcal/day (31.5 ± 6.3 kcal/kg body mass/day) and 2655.7 ± 448.7 kcal/day (33.8 ± 7.2 kcal/kg body mass/day), respectively (Table 4). After taking the supplementation into account, only 11% of the athletes achieved energy recommendations.

The mean daily consumption of dietary carbohydrates by those players who were not taking food supplements and those who were taking food supplements and foodstuffs intended for particular nutritional uses was 364.3 ± 70.0 g/day (4.6 ± 1.0 g/kg body mass/day) and 398.3 ± 81.9 g/day (5.1 g/kg body mass/day), respectively. Fifty-three percent of the players failed to meet carbohydrate recommendations, even after including supplements. The mean daily consumption of fiber was 27.0 ± 8.3 g/day and 29.7 ± 9.9 g/day, respectively (Table 4).

The mean daily consumption of protein by those players who were not taking food supplements and those who were taking food supplements and foodstuffs intended for particular nutritional uses was 113.1 ± 18.0 g/day (1.4 ± 0.3 g/kg body mass/day) and 118.6 ± 20.8 g/day (1.5 ± 0.3 g/kg body mass/day), respectively. The majority of players (65%) reached protein recommendations. After including supplements, 77% of the athletes fulfilled protein recommendations.

The average consumption of fats excluding and including supplements was 70.7 ± 12.1 g/day (0.9 ± 0.2 g/kg body mass/day) and 73.5 ± 70.7 g/day (0.94 ± 0.22 g/kg body mass/day), respectively. Only 38% of the participants met current recommendations for fats (after including supplementations) (Table 4).

Table 5 shows the dietary intake and fulfilment of nutritional recommendations for vitamin and mineral components by studied players (excluding and including supplements). The mean consumption of all the vitamins and mineral components was significantly higher after taking into account all the food supplements and foodstuffs intended for particular nutritional uses. Even with food supplements, the football players did not cover the daily demand for calcium. When supplementation was excluded, the deficiencies of the following vitamins and minerals were observed: folate, vitamin B_2_, vitamin D, vitamin C, vitamin E, and calcium.

It needs to be emphasized that the analyzed diets yielded consumption of potassium and sodium that exceeded the recommended daily intake (Table 5). The diet supplementation further exceeded the daily consumption of sodium and potassium by football players.

## 4. Discussion

Proper nutrition is an essential element of an appropriate motoric preparation of athletes and may be an important factor contributing to an optimal sports outcome. Burke et al. [18] suggest that football players should consume an appropriate quantity of energy necessary to perform physical exercise, increase fat-free body mass, and reduce fat body mass. According to the recommendations, the energy requirement of a player is 40–70 kcal/kg body mass, which corresponds to 2000–7000 kcals/day for a 50–100 kg athlete [19]. During a 90-min football match the player runs an average of 9–13 km and his energy expenditure is 1000–1500 kcal, which is why an appropriate diet is an important component of preparation for the competition period [20]. For elite athletes, energy expenditure during heavy training or competition will further exceed these levels [16].

Our study has shown that the mean energy consumed by the football players including food supplements and foodstuffs intended for particular nutritional uses was 2655.7 ± 448.7 kcal/day (33.8 ± 7.2 kcal/kg body mass). These values are lower than those for soccer players from Netherlands (2988 ± 583 kcal/day; 38.8 ± 7.6 kcal/kg body mass/day) [21], Brazilian professional soccer players (40.74 ± 12.81 kcal/kg body mass/day) [22], Dutch Premier League football players (3285 ± 354 kcal/day; 42.4 ± 3.5 kcal/kg body mass/day) [23], and Greek professional football players (3442 ± 158 kcal/day; 46 ± 2.1 kcal/kg body mass/day) [24]. The energy consumed by the football players we investigated is comparable with the energy consumed by elite youth soccer players from the English Premier League (1927 ± 317 kcal/day; 3.6 ± 7.9 kcal/kg body mass/day) [25], junior teams in the Spanish First Division Soccer League (27943 ± 526 kcal/day; 38.5 ± 8.5 kcal/kg body mass/day) [26], and Australian elite football players (2247 ± 550 kcal/day; 29.7 ± 7.3 kcal/kg body mass/day) [27]. Our results show that the football players we investigated are not consuming enough energy for effective training or that the exercise load of Polish football players may be lower than those in foreign football clubs. It should also be noted that although there is a widespread opinion that athletes can easily replenish their energy by following a well-balanced diet, this is difficult for most athletes, because of the long/high-intensity workouts that are characteristic of the preparation period [16,18]. After an intensive effort, the appetite of the athletes is much lower, while on long training days, the breaks between meals are short, which makes it difficult to consume the correct number of calories. Perhaps it was the lack of appetite and time that caused the studied athletes to consume less energy than the recommendations indicate.

Our study has shown that carbohydrates provided 56.2 ± 4.2% of energy to the football players after including food supplements and foodstuffs intended for specific nutritional purposes. The mean daily consumption of dietary carbohydrates by football players after including food supplements and foodstuffs was 398.3 ± 81.9 g/day (5.1 g/kg body mass/day). The comparison of the obtained results with the norms showed that supplementation did not suffice to cover the recommended intake for the sportsmen training daily for more than 1 h (Table 2) [13]. The problem of low carbohydrate intake in athletes is not limited only to football, but also concerns different team sport disciplines [1]. This point was also clearly highlighted in a review by Burke et al. [18] who demonstrated that carbohydrate needs are largely unmet by high-level athletes. Carbohydrate is a main fuel used for high-intensity aerobic based sports like football and plays a crucial role as a muscle substrate for performance and recovery [28]. Low carbohydrate availability can lead to muscle glycogen depletion and subsequent fatigue, impaired concentration, and decreased physical output [29,30]. It should be noted that promotion of individualized carbohydrate recommendations according to training requirements is important to support training adaptions of athletes [5,29].

The authors’ own studies indicated that the athletes do not periodize carbohydrate consumption (Table 2). The reason for inadequate carbohydrate supply on long/high-intensity training days may be due to physiological factors responsible for appetite [16]. The results of the study indicate that high-intensity physical activity may affect the appetite and the levels of hormones such as ghrelin, glucagon-like peptide 1 (GLP-1), pancreatic polypeptide (PP), and YY peptide (PYYY). The action of these hormones can affect the suppression of hunger after physical activity [31]. Logistical issues, such as time constraints (short breaks between training sessions) and the athletes’ knowledge on how to choose the right food for the main, pre-workout, and post-workout meals can be an additional influence. The foregoing factors may be the reason why athletes do not periodize carbohydrates. Consequently, the education of players and the training staff is extremely important. Both the importance of heeding advice tailored to improve carbohydrate intake and the role that carbohydrates play in refueling trainings and competitions should be highlighted. It is known that carbohydrate periodization has already been implemented to examine its influence on athlete performance in different sports discipline [6,32,33]. The effects of carbohydrate (CHO) periodization in football are still unknown, but one can expect future developments with respect to the subject that will determine whether periodization is helpful, and if so, which manner of it contributes most to successful athletic performance.

The presented study has demonstrated that even in the non-supplemented diet, the protein requirement was met in the diet of the studied athletes. Studies by other authors also indicate that athletes cover their protein needs [21,23] and they often even exceed the normal consumption for this nutrient [15]. Results such as these may be due to the fact that a high-protein diet has been recommended for athletes for many years [34,35]. A proper amount of protein should be ingested daily to guarantee adequate protein synthesis and recovery. Recently, Kerksick et al. [16] suggest that football players’ protein needs range from 1.2 to 1.4 g/kg body mass/day of high quality protein and might be increased to 2.0 g/kg body mass/day for short periods of intensive training, especially during the preparatory period. Current reports indicate that a protein intake above 1.7 g/kg body mass/day does not have a significant impact on the rate of muscle fiber regeneration in the post-workout period, nor will it improve myofibrillar synthesis [3,36]. Having this information in mind, the consumption of doses higher than 2.0 g/kg body mass/day is not required, especially when the athletes have long workouts and the breaks between meals are short, which makes it difficult to consume larger food portions. On the other hand, there are preliminary reports which suggest that protein doses >3 g/kg body mass/day may be beneficial for athletes who require optimization in their body weight composition [15]. However, there is no data on the health effects of the long-term supply of high doses of protein in athletes. The aforementioned recommendations encompass most training regimens and allow for flexible adjustments with periodized training and athletes’ individual needs [13].

In our study, we found that energy from lipids accounted for 25.0 ± 2.9% and 24.3 ± 2.9% of the total energy intake in the case of players who were not taking food supplements and foodstuffs intended for particular nutritional uses and those that were, respectively. Generally, it is recommended that athletes consume a moderate amount of fat (approximately 30% of their daily caloric intake), while proportions up to 50% of daily calories can be safely ingested by athletes during regular high-volume training [16]. Our results showed that football players’ intake of lipids per kilogram of body weight were 0.9 ± 0.2 g/kg body mass/day and 0.94 ± 0.22 g/kg body mass/day, respectively, which in both cases was below the norm [6]. Such a low level of lipid intake is recommended when the main aim is to reduce body fat. Recommendations indicate that the daily dietary fat intake should range from 0.5 to 1 g/kg body mass/day and represent 20% of daily calories [16]. Perhaps the aim of the studied athlete group was to reduce body fat. However, the results of the body mass composition tests obtained by means of electric bioimpedance measurement do not confirm such necessity (Table 1). Completely different results were obtained by researchers who evaluated the fat consumption by athletes in a rugby union [37], in American football [38], and by soccer players [22]. It should be noted that rugby and American football are sports where strength- and speed-based physical activities predominate, rather than the endurance and speed that characterize football. The specificity of the discipline may also determine the supply of macronutrients in the diets of the studied athletes. Fat is a source of energy, fat-soluble vitamins, and essential fatty acids. Although the benefits of fat for exercise performance are equivocal, fat intake is essential for health and appears to maintain the circulation of testosterone concentrations. A diet too low in fats has the potential to compromise health, as it reduces the absorption of fat-soluble vitamins and glycogen storages in muscle [16]. Proper dietary fat amounts and types suggest an immune-modulatory rather than immuno-stimulating effect, as certain parameters of the acute phase response to stress and injury are reduced while other immune system aspects remain intact or enhanced. Furthermore, it is also notable that the natural adaptations to exercise itself, without purposeful omega-3 ingestion or supplementation, increases oleic acid, docosahexaenoic acid (DHA), and total omega-3 fatty acids in the muscle tissue of humans [13,39].

Given the higher exercise load during training, football players have higher requirements for nutrients [16]. Exercise stresses many of the metabolic pathways in which micronutrients are required, and training may result in muscle biochemical adaptations that increase the need for some micronutrients. Athletes often reduce their energy intake, thus eliminating one or more food groups from their diet, which can lead to the intake of an inadequate amount of micronutrients, macronutrients, and vitamins [13]. According to our study, the intake of food supplements and foodstuffs intended for particular nutritional uses by the football players that we investigated resulted in the correction of vitamin B2, folate, vitamin C, and vitamin D. The imbalanced diet and inappropriate intake of vitamins and minerals was also shown in the diets of highly trained endurance athletes [40] and professional soccer players [22]. In our study, despite the use of supplements, the consumption of calcium did not meet the daily requirement. A correct supply of calcium is essential for proper growth, maintenance, and repair of bone tissue and regulation of muscle contraction. In addition, it is responsible for nerve conduction and correct blood coagulation. The risk of fractures due to mechanical injuries is high among athletes. In addition, inadequate calcium intake combined with reduced energy availability increases the risk of lowering bone mineral density, which promotes injury [41,42]. This is why athletes should ensure that they receive an adequate supply of this mineral. If this is not possible through the consumption of calcium-rich foods (allergies to milk proteins; lactose intolerance), a supplementation should be considered.

The results of our study indicated that even without taking supplementation into account, phosphorus and sodium intake in the diets of the studied athletes exceeded the daily recommendations. Similar study results in terms of increased sodium consumption were found in those training in judo [43], CrossFit [44], and soccer [22]. During days of intense training (especially in the heat), a greater amount of water and sodium are lost in sweat. It should be noted that most athletes end their trainings and workouts with a fluid deficit. Nutrition strategies aimed at a rapid return to water balance after physical activity should include adding sodium to food and drinks, thus minimizing diuresis. Thanks to this practice, the amount of fluid retained in an athlete’s body is higher, which promotes post-workout regeneration [45]. Thus, the consumption of sodium by athletes should not be restricted, especially during the summer preparation period [13]. As recommended by Benardot [12], in cases of considerable sodium loss through perspiration, the norm for sodium intake may even exceed 10 g/day.

While the purpose of this study was to estimate the dietary intake of high-rank Polish football players, it possesses limitation. The authors of the study tried to minimize the risk of improperly completed nutrition logs by instructing the athletes on how to fill them out correctly and were in constant contact with players. Nevertheless, potential limitations of the study include reliability and validity of the estimation of the average food intake, which were based on subjective assessments by the subjects. This may have resulted in under-reporting of total energy and nutrient intake by the studied players [46,47].

## 5. Conclusions

This study provides insight into the dietary intake of high-rank professional Polish football players during a preparatory period. Our results showed that players did not meet recommendations for energy, vitamin B_2_, vitamin C, vitamin E, folate, and calcium and exceeded recommendations for sodium and potassium intake. The results of this study do not confirm the justification for adding protein preparations to the diets of the studied players. Furthermore, the football players’ dietary carbohydrate intake was relatively low in comparison to requirements based on training loads. Based on our results we conclude that further work is necessary to reinforce education in this area in order to enhance athletic performance by the optimization of glycogen storage and resynthesis. Moreover, sports nutritionists working with elite football teams need to use an individualized approach when periodizing an athlete’s carbohydrate intake to support athletes’ training and match daily nutrition goals.

## Figures and Tables

**Table 1:** Participants’ characteristics.

Characteristic	Mean ± SD (*n* = 26)
Age (years)	27.0 ± 3.7
Body weight (kg)	78.3 ± 6.9
Height (m)	1.9 ± 0.1
Career duration (years)	14.7 ± 4.2
TBW (liters|%)	47.2 ± 4.6|58.8 ± 2.6
Body fat (kg|%)	15.6 ± 3.1|19.5 ± 3.3
FFM (kg|%)	64.3 ± 6.4|80.4 ± 3.5
Muscle mass (kg|%)	47.0 ± 4.8|58.8 ± 3.4
VO_2peak_ (ml/kg/min)	55.8 ± 4.0

TBW: total body water; FFM: fat free body mass; VO_2peak_: peak oxygen uptake.

**Table 2:** Training loads during one week of the preparatory period and comparison of carbohydrate targets to carbohydrate intake by studied players.

Training Loads		Duration (min)	CHO [6]	CHO Intake (g/kg Body Mass/Day) (Mean ± SD)	Athletes Scoring Below Required Norm (%)
Day 1	**Morning**: Physical training: strength and power training	90	6–10 g/kg body mass/day (~1–3 h/day moderate to high intensity exercises or multiple session training day)	4.7 ± 1.4	76.9
	**Afternoon**: Technical, tactical session	110			
Day 2	**Morning**: Physical training: strength training	90	5–7 g/kg body mass/day for moderate exercise (~1 h/day)	5.3 ± 1.3	57.7
Day 3	**Morning**: Physical training: endurance (interval training)	120	5–7 g/kg body mass/day for moderate exercise (~1 h/day)	4.8 ± 1.4	46.2
Day 4	**Morning**: Physical training: endurance (interval training)	120	6–10 g/kg body mass/day (~1–3 h/day moderate to high intensity exercises or multiple session training day)	5.4 ± 1.5	53.9
	**Afternoon**: Technical training	90			
Day 5	**Morning**: Physical training: strength, power, and speed training	90	5–7 g/kg body mass/day for moderate exercise (~1 h/day)	5.2 ± 1.5	46.2
Day 6	**Afternoon**: Game	120	6–10 g/kg body mass/day (~1–3 h/day moderate to high intensity exercises or multiple session training day)	6.1 ± 1.8	42.3
Day 7	Off	–	3–5 g/kg body mass/day rest day	5.3 ± 1.5	26.9

CHO: carbohydrates.

**Table 3:** Supplements used by the players including serving size.

Supplement	Serving
Carbohydrate	0.6–1.2 g/kg body mass (after a training session)
Creatine	5 g/day
Energy gel	Self-directed
Isotonic drink	Self-directed
Multivitamin	Self-directed
Vitamin D	50–100 µg/day
Sports bar	Self-directed
Whey protein isolate	20–30 g (after a training session)

**Table 4:** Energy and macronutrient intake data collected via a 7-day food diary.

Component	Recommendations	Diet Excluding Supplements (Mean ± SD)	Diet Including Supplements (Mean ± SD)	*P*
**Energy**				
(kJ/day)	–	10,376.4 ± 1620.7	11127 ± 1881.1	0.0000
(kcal/day)	3400–4300 [12]	2480.3 ± 388.6	2655.7 ± 448.7	0.0000
(kcal/kg body mass/day)	47–60 [12]	31.5 ± 6.3	33.8 ± 7.2	0.0000
**Carbohydrates**				
(g/day)	–	364.3 ± 70.0	398.3 ± 81.9	0.0000
(g/kg body mass/day)	5–12 [13]	4.6 ± 1.0	5.1 ± 1.2	0.0000
**Fiber** (g/day)	20–40 [14]	27.0 ± 8.3	29.7 ± 9.9	0.0000
**Protein**				
(g/d)	–	113.1 ± 18.0	118.6 ± 20.8	0.0002
(g/kg body mass/day)	1.2–2.0 [15]	1.4 ± 0.3	1.5 ± 0.3	0.0002
**Fat**				
(g/d)	–	70.7 ± 12.1	73.5 ± 13.1	0.0014
(g/kg body mass/day)	1–1.5 [16]	0.90 ± 0.20	0.94 ± 0.22	0.0015
(% Energy)	20–35 [13]	25.0 ± 2.9	24.3 ± 2.9	0.0003

**Table 5:** Mineral and vitamin intake by football players.

Vitamin/Mineral	Recommendation (12)	Diet Excluding Supplements (Median/Mean ± SD)	Diet Including Supplements (Median/Mean ± SD)	*P*
Vitamin B_1_ (mg/d)	1.5–3.0	1.8	2.3	0.0006
Vitamin B_2_ (mg/g)	1.1/1000 kcal	2.2 ± 0.4	3.1 ± 1.1	0.0021
Niacin (mg/d)	14–20	28.9 ± 6.0	34.9 ± 8.6	0.0003
Vitamin B_6_ (mg/d)	1.6–2.0	2.7	3.6	0.0001
Vitamin B_12_ (μg/d)	2.4–2.5	5.9	6.6	0.0001
Folate (μg/d)	400	354.4 ± 86.8	433.9 ± 121.4	0.0000
Vitamin A (μg/d)	700–900	913.8 ± 254.4	1609.6 ± 691.3	0.0000
Vitamin D (μg/d)	20–50 [17]	4.9	56.5	0.0000
Vitamin C (mg/d)	200	191.9 ± 71.1	255.7 ± 120.2	0.0005
Vitamin E (mg/d)	15	11.1 ± 4.6	16.0 ± 6.7	0.0000
Calcium (mg/d)	1300–1500	1179.9 ± 265.8	1291.5 ± 318.2	0.0000
Phosphorus (mg/d)	1250–1500	1881.6 ± 355.0	1969.8 ± 402.4	0.0000
Magnesium (mg/d)	400–450	469.5 ± 114.3	519.3 ± 136.3	0.0000
Potassium (mg/d)	2000	4510.8 ± 780.0	5019.8 ± 1035.1	0.0000
Sodium (mg/d)	1500	3889.6 ± 606.3	4362.6 ± 739.4	0.0000
Iron (mg/d)	15–18	14.9 ± 2.5	16.7 ± 3.3	0.0000
Zinc (mg/d)	11–15	13.3 ± 2.4	14.9 ± 3.4	0.0007
Iodine (μg/d)	150	158.2 ± 32.6	177.0 ± 42.2	0.0000
